# Genome-Wide Variation Profile of the Genus *Tobamovirus*

**DOI:** 10.3390/v17091284

**Published:** 2025-09-22

**Authors:** Amany E. Gomaa, Hernan Garcia-Ruiz

**Affiliations:** 1 Department of Plant Pathology and Nebraska Center for Virology, University of Nebraska-Lincoln, Lincoln, NE 68583, USA; amanyateaa@mans.edu.eg; 2 Department of Botany, Faculty of Science, Mansoura University, Mansoura 35516, Egypt

**Keywords:** *Tobamovirus*, nucleotide diversity, selection pressure, phylogenetic analysis, Tobamovirus fructirugosum, resistance breaking, ToBRFV

## Abstract

The genus *Tobamovirus* belongs to the family *Virgaviridae*, and the genome consists of monopartite, positive, single-strand RNA. Most species contain four open reading frames encoding four essential proteins. Transmission occurs primarily through mechanical contact between plants, and in some cases, via seed dispersal. *Tobamovirus fructirugosum* (tomato brown rugose fruit virus, ToBRFV), the most recently described species in the genus, was first reported in 2015. It overcame genetic resistance that had been effective in tomato for sixty years, causing devastating losses in tomato production worldwide, and highlights the importance of understanding *Tobamovirus* genomic variation and evolution. In this study, we measured and characterized nucleotide variation for the entire genome and for all species in the genus *Tobamovirus.* Additionally, we measured the selection pressure acting on each open reading frame. Results showed that low nucleotide diversity and negative selection pressure are general features of tobamoviruses, with values that are approximately the same across open reading frames and without hypervariable areas. A comparison of nucleotide diversity between *T. fructirugosum* and its close relatives, *T. tomatotessellati* (tomato mosaic virus, ToMV) and *T. tabaci* (tobacco mosaic virus, TMV), showed low nucleotide diversity in the movement protein region harboring the resistance-breaking mutation. Furthermore, phylogenetic and diversity analyses showed that *T. fructirugosum* continues to evolve, and geographical distribution and host influence genomic diversity.

## 1. Introduction

The genus *Tobamovirus* (tobamoviruses) belongs to the family *Virgaviridae* and includes several species that cause important diseases in crops [1,2], such as *Tobamovirus fructirugosum* (tomato brown rugose fruit virus, ToBRFV), a species first described in 2015 that broke *Tm*2^2^-mediated resistance to tobamoviruses in tomato [3,4]. General features of tobamoviruses include monopartite, positive, single-strand RNA; rigid, rod-shaped particles [5]; transmission through mechanical means and seed contamination; long-term persistence in soil within infected plant residues [6]; and transmission by pollinator insects [7,8].

**Figure 1:**
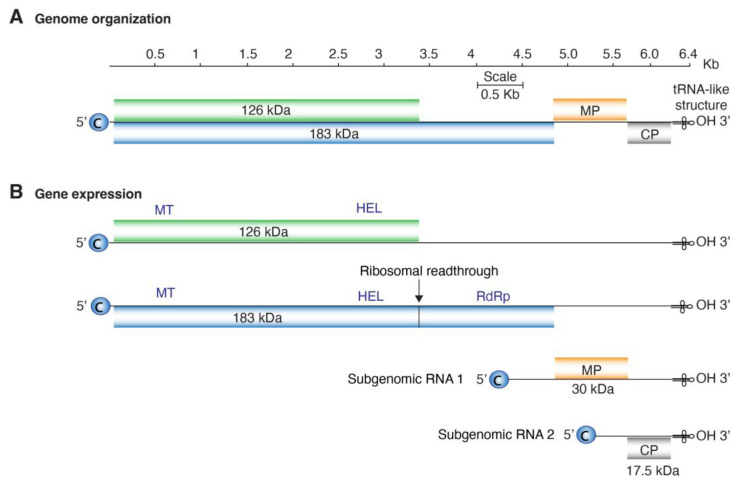
*Tobamovirus* genome organization and gene expression. (**A**) Map showing genome organization of the four main ORFs in *T. tabaci* (tobacco mosaic virus, TMV accession number NC_001367.1). Single lines represent non-coding regions, and labeled boxes represent open reading frames. (**B**) Gene expression strategies include the formation of sub-genomic RNAs and ribosomal readthrough. Functional methyltransferase (MT), helicase (HEL) and RNA-dependent RNA polymerase (RdRp) domains for 126 kDa and 183 kDa proteins are indicated.

The tobamoviral genome consists of approximately 6400 nucleotides [9]. The genome is organized into four core open reading frames (ORFs), with two additional ORFs described in some species (Figure 1). The genomic RNA expresses a 126 kDa protein that contains a methyltransferase and a helicase domain [10,11,12]. The second ORF encodes a 183 kDa protein, produced by readthrough of the 126 kDa termination signal, and includes an RNA-dependent RNA polymerase (RdRp) domain. The movement protein and coat protein are expressed from subgenomic RNAs [5,13]. Two additional proteins have been described for some species, including *T. tabaci* (tobacco mosaic virus, TMV): a 54 kDa protein corresponding to the RdRp domain of the 183 kDa protein, and a small 4–6 kDa protein (P6 protein) also known as charged protein [14]. The length and overlap of the open reading frames vary among species, and the size of the replicase protein varies between strains, even within the same species [14,15].

Recombination has been documented for several species and plays an important role in *Tobamovirus* evolution and host adaptation [16]. Natural recombination has occurred in the replicase, movement protein, and coat protein genes, forming chimeras between tobacco mosaic virus (TMV) and tomato mosaic virus (ToMV) as well as recombinant isolates of tobacco mild green mosaic virus (TMGMV) [17].

Breeding for genetic resistance to tobamoviruses has been a valuable management practice [5]. *T. fructirugosum* (ToBRFV) was first detected in Israel and Jordan in 2014 and 2015 [3,4], and has spread globally [18]. According to the current working model, ToBRFV emerged from a recombination event between *T. tomatotessellati* (tomato mosaic virus, ToMV) and *T. tabaci (*tobacco mosaic virus, TMV) [4]. After selection and adaptation, ToBRFV broke *Tm*2^2^-mediated resistance to tobamoviruses in tomato that had been effective for sixty years [3,4], causing widespread economic losses and becoming a major threat to tomato production globally [19]. The emergence of ToBRFV highlights the need to better understand genomic diversity and evolutionary mechanisms of tobamoviruses [20]. Moreover, the global dissemination of tobamoviruses through trade and seed distribution has contributed to their rapid spread, making it imperative to study the geographical and temporal dynamics of their populations [20,21].

Previous studies have focused on the identification of genomic variation within individual tobamoviral species in particular areas of the world or within areas of the genome [21,22,23,24]. In this study, we took a general, genome-wide, and comparative approach to measure genomic variation for the entire genus *Tobamovirus*. We also measured selection pressures on the top five most variable species (based on nucleotide diversity) to identify deleterious mutations that reduce viral fitness and are removed by negative (purifying) selection, as well as advantageous mutations that provide a competitive advantage such as viral replication, movement, or immune evasion, and are maintained by positive selection [25]. In the second part of the study, we focused on the global evolution patterns of ToBRFV due to its current economic impact. Results identified key characteristics of tobamovirus diversity and evolution. Nucleotide diversity and selection patterns across species showed that low genomic variation, negative selection pressure, and lack of hypervariable areas are general features of tobamoviruses, including ToBRFV. However, influenced by geographical distribution and hosts, ToBRFV is still mutating and evolving into new strains due to variation at the single amino acid level instead of specific regions at the protein or domain levels.

## 2. Materials and Methods

All computational analyses were performed using a high-performance computing node system available through the Holland Computing Center (HCC) at the University of Nebraska-Lincoln. The in-house Python (3.12.5), Bash (5.2), and R scripts used are available upon request.

### 2.1. Tobamovirus Genomic Sequences

Genomic sequences for all species in the genus *Tobamovirus* were retrieved from the National Center for Biotechnology Information (NCBI) database on 18 June 2024, using a customized Python script incorporating Batch Entrez and SeqI0 based on Entrez Programming Utilities. For each species, a reference genome was chosen (Table 1). If NCBI had an officially defined reference genome, it was used; otherwise, the longest nucleotide sequence was selected. For each species, the reference genome was used to determine the ORF coordinates. All downloaded accessions were filtered using a Bash script to remove partial sequences and retain only complete genomes and accessions measuring at least 95% the length of the reference genome. Accessions with less than 95% of the reference genome length were excluded, and accessions containing more than 2.5% unknown characters were also excluded. In *Tobamovirus* taxonomy, the primary criterion for species demarcation is a nucleotide difference of at least 10%. Accordingly, if two tobamoviruses have more than 90% whole-genome nucleotide sequence identity, they are considered strains of the same species. Conversely, if they have less than 90% sequence identity, they are considered different species [26]. Thus, for each species, accessions were compared to the reference accession and discarded if their nucleotide identity was below 90% [27]. Based on these criteria, accessions representing chimeric viruses resulting from RNA recombination were removed from the dataset. To ensure robust statistical comparisons, variation analyses were conducted only for species with a minimum of three accessions meeting the criteria described above [28]. The final number of sequences retained after these filtering steps is indicated in Table 1. There are differences in tobamovirus species recognized by ICTB and NCBI taxonomy. Thus, in addition to the ICTV-recognized species, our analysis also included some unclassified tobamoviruses listed by NCBI taxonomy. Unclassified species with complete genomes are indicated in Table 1 and represent viruses that have been sequenced, such as watermelon green mottle mosaic virus (WGMMV), but are not yet formally assigned species status by ICTV. WGMMV is closely related to but different from *T. viridimaculae* [29]. Accessions used in the analyses for all species are indicated in Supplementary Table S1.

### 2.2. Tobamovirus Phylogeny

Consensus sequences were generated for each virus species using the genome sequences that passed the filters indicated above. Consensus sequences were then combined into a single FASTA file and aligned using MAFFT version 7.4 (Multiple Alignment using Fast Fourier Transform). A neighbor-joining phylogenetic tree was constructed from the aligned sequences, and the resulting alignment was exported in Newick format. The phylogenetic tree was visualized using Figtree version 1.4.4 http://tree.bio.ed.ac.uk/software/figtree/, accessed on 10 January 2025 [30].

### 2.3. Single-Nucleotide Polymorphism and Nucleotide Diversity

For each virus species with more than three complete accessions, alignment files (.aln) based on forward reads from MAFFT were analyzed for single-nucleotide polymorphism (SNPs) using bash scripts as described in [31]. SNPs were normalized across the genome using a 50 nt sliding window. Average pairwise nucleotide diversity (π = Pi) was calculated using TASSEL version 5.0 [32] from alignment files (.aln) for each species, also employing a 50 nt sliding window.

Nucleotide diversity captures variation at the population level by normalizing the number of polymorphic sites to the number of accessions [33]. Thus, based on nucleotide diversity, the five most diverse viruses were selected for further analyses to map the genomic distribution of variation and for selection analyses and their genomic distribution. Nucleotide diversity for each viral open reading frame (ORF) was estimated by calculating the weighted average of the Pi values from the sliding windows within each gene. This was carried out by summing the Pi values for all windows covering the gene, multiplying each by the window length (50 nt), and then dividing by the total gene length [34]. *T. fructirugosum* and its ancestors *T. tabaci* and *T. tomatotessellati* [4] were also included in the analyses due to their current importance. For both SNPs and Pi, the mean values along with the 99% confidence intervals (*p*-value < 0.01) were estimated, and a genome-wide map was obtained for each species.

### 2.4. Selection Analyses

For each species, accession numbers were used to retrieve coding sequences (CDSs) from the NCBI database using Batch Entrez. A custom Bash script was used to separate CDSs for each gene. The stop codon in the ribosomal read-through region of the 126 kDa was removed using a Python script to obtain the complete sequence for the 183 kDa replicase gene. The rates of synonymous substitutions (dS) and non-synonymous substitutions (dN) [35] were calculated for each gene’s CDS using the SLAC (Single-Likelihood Ancestor Counting) and MEME (Mixed Effects Model of Evolution) tools on Datamonkey (https://www.datamonkey.org, last accessed on 10 January 2025). A significance threshold of ≤0.05 and a posterior probability of >0.95 were applied for both SLAC and MEME [31,36]. The dN/dS ratio was calculated, and a selection pressure map for the entire genome was created by stitching the ORFs together. The expected and observed numbers of sites under selection were calculated using the model previously described in [31,37,38]. In this model, the expected number of sites under positive or negative selection for each ORF was estimated by dividing the length of the ORF by the total length of the genome, then multiplying this proportion by the total number of sites under negative or positive selection genome-wide. This expected value was then compared to the observed number of sites in each ORF.

### 2.5. Maximum Likelihood Phylogenetic Tree for T. fructirugosum

We constructed a maximum likelihood phylogenetic tree for the alignment sequences of all available *T. fructirugosum* in NCBI using IQtree (http://www.iqtree.org, last accessed on 1 March 2025). The accessions used are listed in Table S2. The tree was subjected to 1000 bootstrap replicates [39]. The best-fit nucleotide substitution model was determined using ModelTest [40]. The resulting tree was visualized with a custom R script.

### 2.6. Multidimensional Scaling

To investigate the influence of geographical distribution, host, and year of isolation on the variation in *T. fructirugosum*, we used multidimensional scaling (MDS) [41] to visualize the virus sequences in two-dimensional Euclidean space. MDS, a non-linear dimensionality reduction technique, transforms a pairwise distance matrix into a lower-dimensional representation while best recapitulating the original distance matrix. The distance matrix was generated from the alignment file containing all complete *T. fructirugosum* accessions as the input using the dist.dna function from the dplyr package in R. MDS analysis and visualization were performed with a custom R script.

## 3. Results

### 3.1. Tobamovirus Group According to the Botanical Family of Their Host

**Figure 2:**
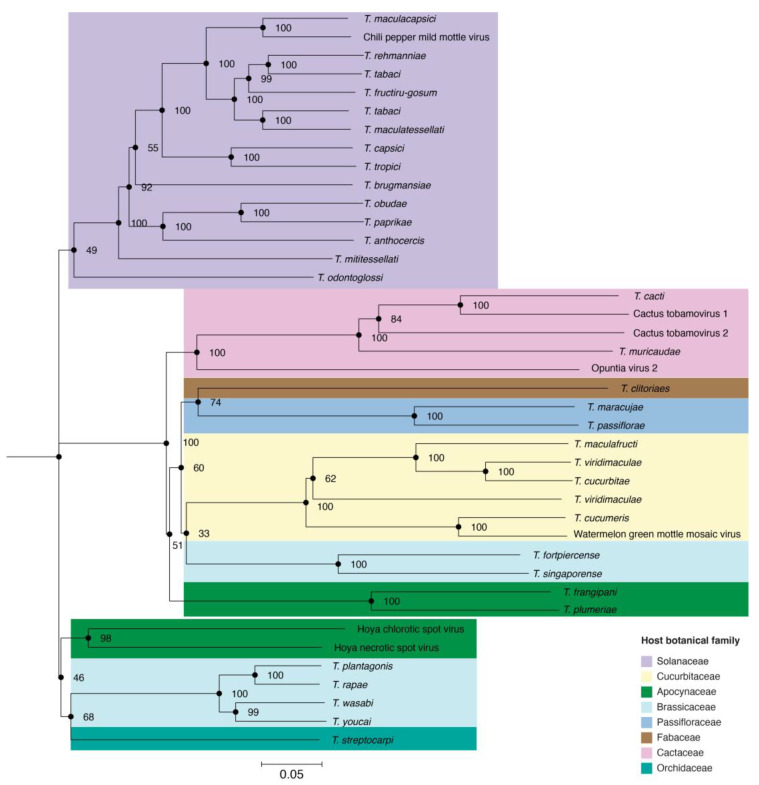
*Tobamovirus* phylogeny as inferred from consensus nucleotide sequences from all accessions in Table 1 that were ≥95% of the reference accession length and species with at least two complete genomes. Consensus sequences were aligned using MAFFT, and the phylogenetic tree was generated using FigTree v1.4.4. Colors in the tree indicate the botanical family of the primary host. Branches are labeled to indicate bootstrap values based on 1000 replicates. Binomial names are used for classified species; virus names are used for species not classified by ICTV.

Genetic relationships among *Tobamovirus* species were determined by constructing a phylogenetic tree based on nucleotide sequences. All species with at least two complete genomes were included. The resulting tree comprised thirty-three classified species and seven unclassified species, which clustered into three clades. The botanical family of the hosts was added to each virus species (Figure 2). The first clade was formed by tobamoviruses that predominantly infect plants in the family *Solanaceae*. In the second clade, more than half of the species mainly infect plants in the families *Cucurbitaceae* and *Cactaceae*.

The third clade is dominated by species that infect plants in the family *Brassicaceae*. This phylogenetic organization highlights that sequence similarity between *Tobamovirus* species correlates with the botanical family of their host and indicates that host selection pressure is a determinant of virus adaptation.

### 3.2. Tobamovirus Genome Diversity

**Figure 3:**
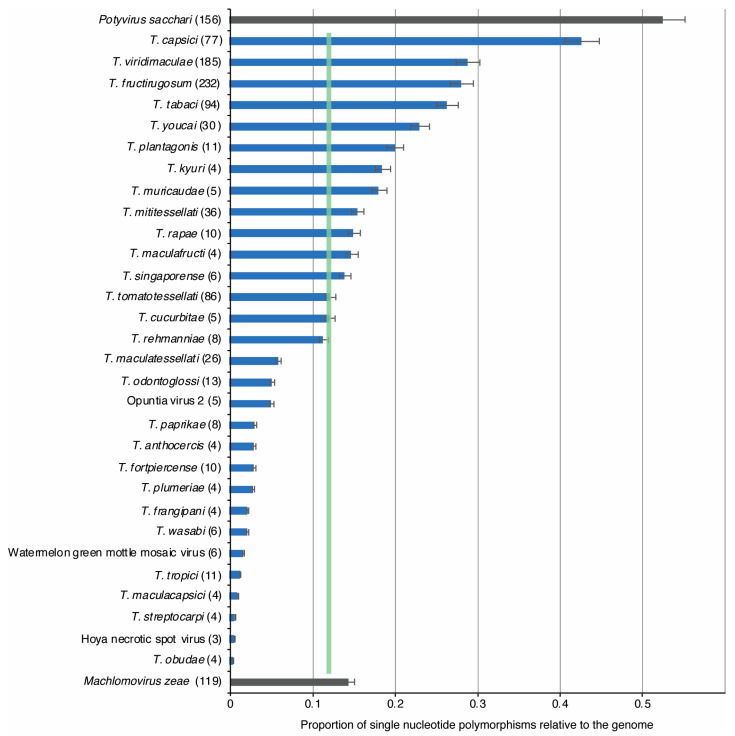
Genomic variation in tobamoviruses. For species with three or more accessions, genomic variation was determined as single-nucleotide polymorphisms relative to the genome and estimated in a 50 nt window. Bars represent the average and standard error of the proportion of polymorphic sites (number of single-nucleotide polymorphisms/length of the genome). For each species, the number of nucleotide accessions is indicated in parentheses. A green vertical line represents the mean and a 99% confidence interval (*p*-value < 0.01). Species binomial names indicate classified tobamoviruses, while viruses with common names were unclassified. For comparison, *Potyvirus sacchari* (sugarcane mosaic virus) and *Machlomovirus zeae* (maize chlorotic mottle virus) were used as hypervariable and genetically stable, respectively [31].

Using a 50-nucleotide window, genomic diversity in the genus *Tobamovirus* was assessed by comparing single-nucleotide polymorphism (SNP) relative to genome length (genomic variation) for all species with three or more complete genome sequences [31]. Highly variable *Potyvirus sacchari* (sugarcane mosaic virus, SCMV) and low-diversity *Machlomovirus zeae* (maize chlorotic mottle virus, MCMV) [42] were included as internal controls for comparison. Results showed that the average genomic variation in tobamoviruses is 0.11 ± 0.2 (Figure 3), which means that approximately 11% of positions in the genome are polymorphic. This level of genomic variation is 4.8 times lower than that observed for *P. sacchari* and approximately the same (1.1 times) as that observed for *M. zeae* (Figure 3).

Tobamoviruses with the highest genomic variation were *T. capsici*, *T. viridimaculae*, *T. fructirugosum*, and *T. tabaci*. While all tobamoviruses have lower genomic variation than *P. sacchari*, 18 tobamovirus species showed lower genomic variation than *M. zeae* (Figure 3). These results show that low genomic variation is a general feature of tobamoviruses.

**Figure 4:**
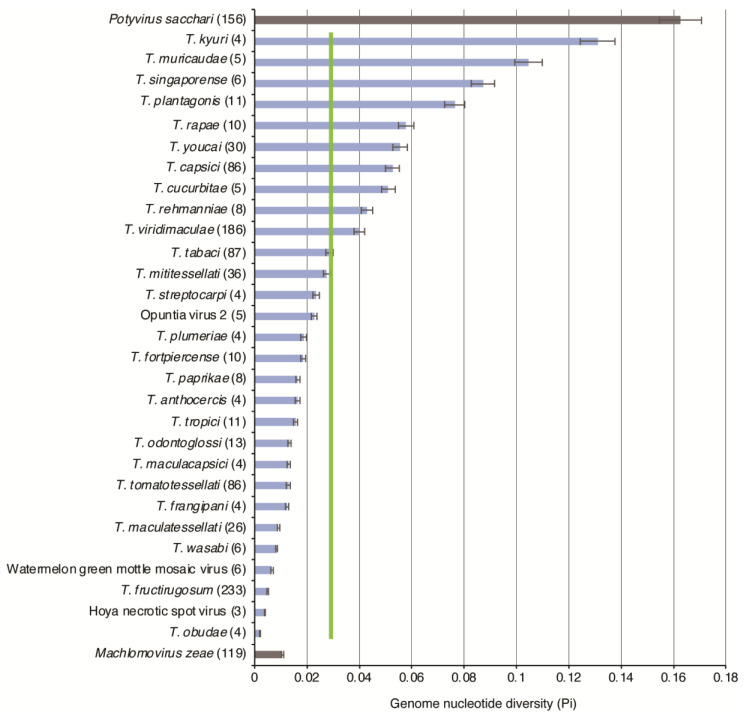
Nucleotide diversity (Pi) in tobamoviruses. Species with three or more accessions were included. Bars represent the proportion of variable positions with respect to the length of the genome and are normalized to the number of accessions. Labels are as in Figure 3.

As a complementary approach, nucleotide diversity (Pi) was used to measure genetic variation in tobamoviruses. Nucleotide diversity accounts for both the number of polymorphic sites and the frequency of different nucleotides at each site, while also normalizing variation to the number of accessions [31,43]. Nucleotide diversity was calculated using Tassel software Version 5.0 [28,31]. Results show that the average nucleotide diversity for tobamoviruses is 0.03, which is 5.5 times lower than that observed for *P. sacchari* and only 2 times higher than that observed for *M. zeae* (Figure 4). The five most diverse species were *T. kyuri*, *T. muricaudae*, *T. singaporense*, *T. plantagonis*, and *T. rapae* (Figure 4). These species were chosen for further variation analysis. *T. fructirugosum* was included due to its current importance.

Collectively, using two complementary approaches, the results described above show that low genomic variation is a general feature of tobamoviruses (Figure 3 and Figure 4).

### 3.3. Nucleotide Diversity by Open Reading Frame

**Figure 5:**
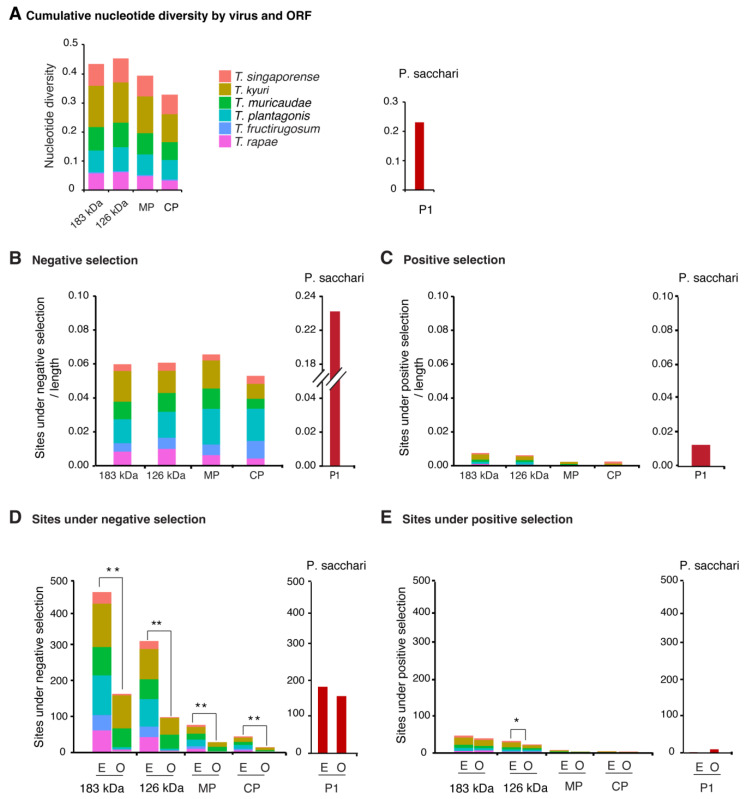
Nucleotide diversity and selection by open reading frame for the top five most variable tobamoviruses and ToBRV. Parameters were estimated per ORF and normalized to the corresponding gene length. Virus names are color-coded. *Potyvirus sacchari* P1 was used as a control [31]. (**A**) Cumulative nucleotide diversity across ORFs for each virus species. No significant differences in nucleotide diversity were detected among the four ORFs (Chi-square test, *p*-value = 0.99). (**B**) Number of sites under negative selection normalized to ORF length. (**C**) Number of sites under positive selection normalized to ORF length. (**D**) Expected (E) and observed (O) number of sites under negative selection. Expected values were determined assuming a random distribution in the genome. (**E**) Expected and observed number of sites under positive selection. The * denotes significant differences with *p*-value ≤ 0.5, ** for *p*-value ≤ 0.001, as calculated using the Chi-square test.

We used nucleotide diversity to measure variation per open reading frame (ORF) for the five most variable species and *T. fructirugosum* (Figure 5A). No significant differences were detected among the four core open reading frames. The highest nucleotide diversity was observed in the ORF encoding the 126 kDa protein, with an average value of 0.1. For comparison, the P1 ORF of *Potyvirus sacchari* exhibited a nucleotide diversity of 0.22, which is 2.2 times higher.

### 3.4. Selection Analysis by Open Reading Frame

The ratio of non-synonymous to synonymous substitutions (dN/dS) is widely used to determine how natural selection affects the evolution of viral populations [44]. A dN/dS ratio less than 1 indicates negative selection pressure, where harmful mutations are eliminated to preserve essential gene functions. A ratio equal to 1 reflects neutral selection, where mutations have no effect on fitness and accumulate randomly. A ratio greater than 1 indicates positive selection, where changes in the gene sequence are beneficial and favored, allowing the virus to adapt to different hosts or overcome immune defenses [45,46]. For comparison, we estimated negative and positive selection in *P. sacchari* P1. The proportion of sites under negative selection in tobamoviruses, normalized by ORF length, was 0.01 on average, which was approximately 23 times lower than that observed in *P. sacchari* P1 (Figure 5B). Positive selection was barely above background and approximately 10 times lower than that observed in *P. sacchari* P1 (Figure 5C). Within tobamoviral ORFs, sites under negative selection occurred at a frequency six times higher than those under positive selection (Figure 5B,C). No significant differences in positive or negative selection were observed among the four core ORFs of tobamoviruses (Figure 5B,C).

The frequency of sites under positive or negative selection was compared to the frequency expected randomly. Results showed that in all open reading frames, the observed frequency of sites under negative selection was significantly lower than that expected randomly (*p* ≤ 0.001) (Figure 5D). Positive selection was low across the genome, with only the ORF encoding the 126kDa protein showing a higher expected than observed frequency (Figure 5E). Collectively, these results indicate that tobamoviruses are under negative selection.

### 3.5. No Hypervariable Areas Were Detected in the Tobamovirus Genome

**Figure 6:**
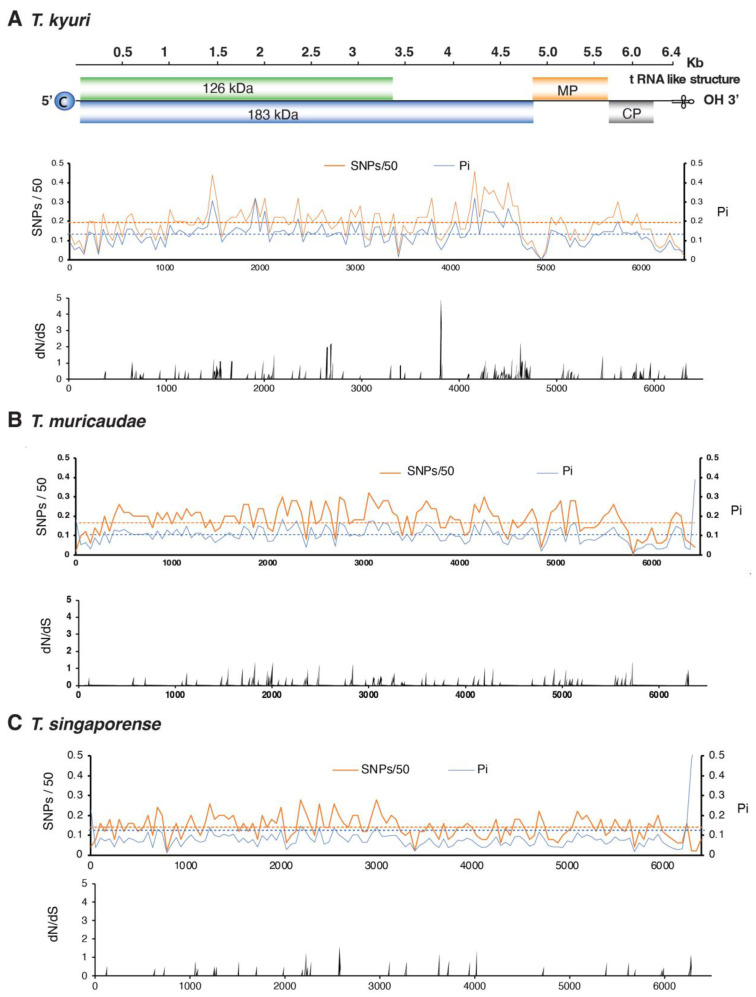
Genome-wide variation in the tree most variable tobamoviruses. Single-nucleotide polymorphism (SNP), nucleotide diversity (Pi), and the ratio of non-synonymous to synonymous changes (dN/dS) were estimated in a 50 nt window. The average and a 99% confidence interval (*p*-value < 0.01) are indicated as a horizontal line. ORFs are color-coded and labeled with the corresponding protein names. Genome coordinates are based on the following reference accessions: (**A**) *T. kyuri* (NC_003610.1); (**B**) *T. muricaudae* (NC_016442.1); (**C**) *T. singaporense* (NC_008310.2).

**Figure 7:**
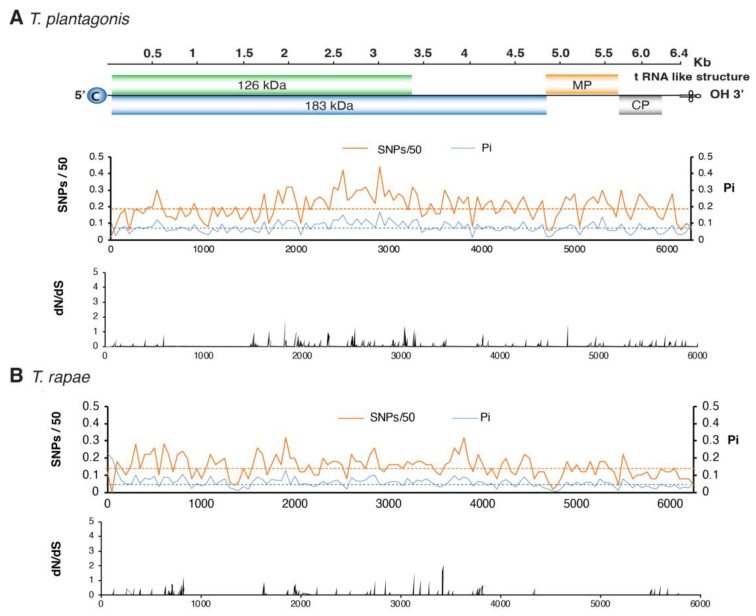
Genome-wide variation in the fourth and fifth most variable tobamoviruses. Labels are as described in Figure 6. Genome coordinates are based on the following reference accessions: (**A**) *T. plantagonis* (NC_002792.2); (**B**) *T. rapae* (NC_001873.1).

Genome-wide variation in the five most variable species was analyzed to identify whether variation concentrates in areas of the genome or is randomly distributed. The ratio of non-synonymous to synonymous substitutions (dN/dS) was estimated in a 50-nucleotide (nt) window to identify the region that accumulates mutations in the genome. No consistently hypervariable regions were detected across species (Figure 6 and Figure 7).

### 3.6. Variation in T. fructirugosum

**Figure 8:**
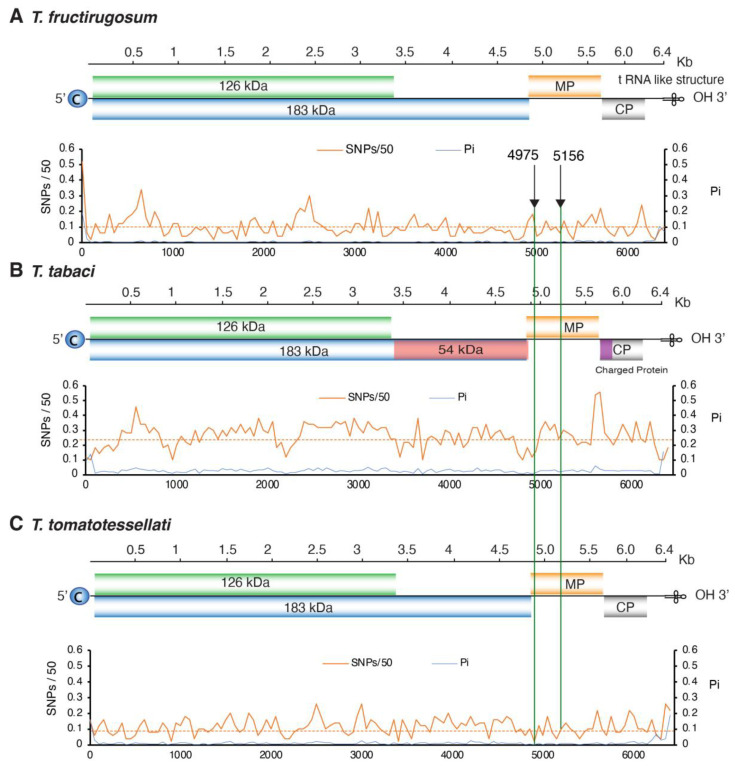
Genome-wide variation in *T. fructirugosum* and its closest relatives. (**A**) *T. fructirugosum.* Black arrows indicate resistance-breaking mutations in the movement protein (MP) of ToBRFV at nucleotide positions 4975 and 5156. At both sites, T is substituted by A in strain ToBRFV-Tom2M-Jo-MZ2438228.1, resulting in amino acid changes at amino acids 22 (Phe to Tyr) and 82 (Asn to Lys) [47], while in strain ToBRFV_G78_RB-OR760199, at position 5156, T was replaced by G, leading to an Asn to Lys mutation in amino acid 88 [47,48]. (**B**,**C**) Green lines mark the equivalent positions of MP in (**B**) *T. tabaci* and (**C**) *T. tomatotessellati,* corresponding to the resistance breaking sites identified in *T. fructirugosum* strains.

While there is low genomic variation and predominantly negative selection across the entire genus *Tobamovirus*, *T. fructirugosum*, the most recently identified species, has broken resistance in tomato varieties due to variation in a single amino acid within the movement protein. Similar mutations occurred independently twice in two different strains [47,48]. These positions map to nucleotides 4975 and 5156 [47,48], corresponding to nucleotide changes from thymine (T) to adenine (A) at the two positions. In ToBRFV-Tom2M-Jo-MZ2438228.1, these nucleotide substitutions resulted in amino acid changes at position 22 (change from Phe to Tyr) and position 82 (change from Asp to Lys) in the movement protein, respectively (Figure 8) [47]. In strain ToBRFV_G78_RB-OR760199, nucleotide 5156 mutated from T to guanine (G), resulting in an amino acid change from Asn to Lys at position 82 of the MP [48]. In *T. fructirugosum* and its closest relatives, *T. tabaci* and *T. tomatotessellati,* this region of the movement protein does not accumulate mutations at a particularly high rate (Figure 8).

### 3.7. Phylogenetic Analysis of T. fructirugosum

**Figure 9:**
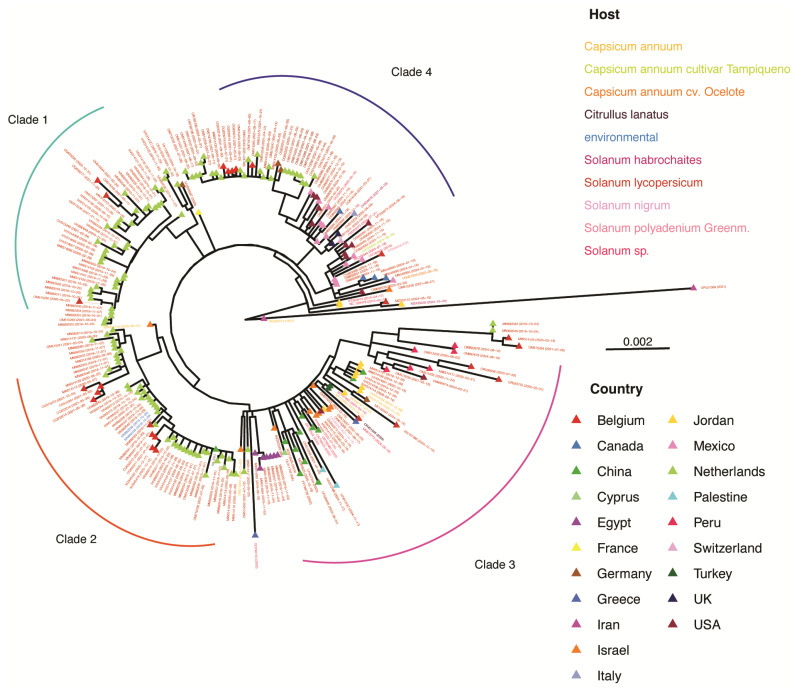
Maximum-likelihood phylogenetic tree of ToBRFV complete genome accessions constructed using IQ-TREE with 1000 bootstrap replicates. The country of origin of each isolate is represented by colored triangles, while the host species is indicated by the color of each accession label. The four main clades are labeled. Accessions used are listed in Supplementary Table S2.

To gain insight into the evolution of *T. fructirugosum* (ToBRFV), a phylogenetic tree was generated from all available complete genome accessions using the maximum likelihood model in IQ-tree [39]. Results indicate the presence of four clades (Figure 9). The country of origin of each accession was mapped in the tree. One clade is dominated by accessions from the Netherlands, another by accessions from the Middle East and China, and a third by accessions from North America. Most of the accessions were isolated from *Solanum sp.* and *Capsicum sp.* Interestingly, differences in host affect the phylogeny of ToBRFV, suggesting that the virus has mutated and evolved to adapt to each host. The year of isolation for each accession was also mapped, showing that isolates from the same year tend to cluster together.

### 3.8. Geographical Distribution Correlates with Virus Variation

**Figure 10:**
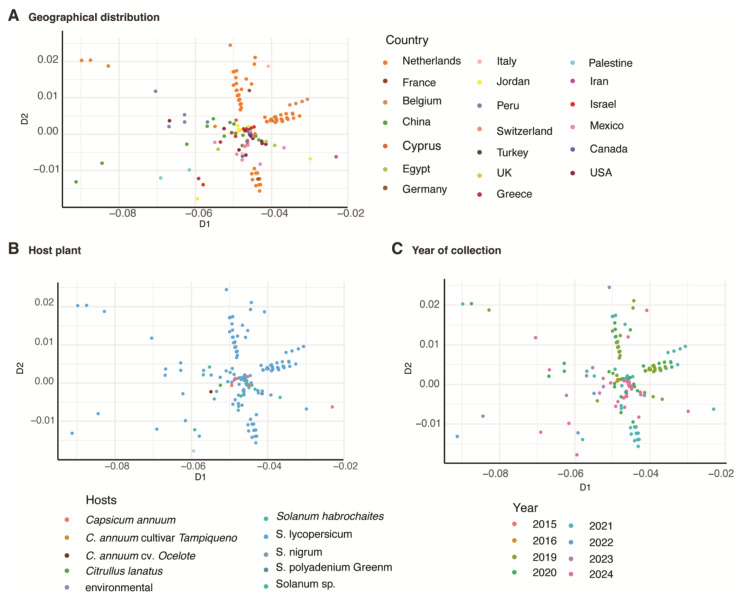
Multidimensional scaling clustering generated from the genomic distance matrix of 232 ToBRFV accessions. (**A**) Diversity based on geographical distribution; the color of circles represents the origin country of accessions. (**B**) Diversity based on host plant. (**C**) Diversity based on the year of collection for each accession.

As indicated above, the phylogenetic organization of *ToBRFV* accessions appears to be influenced by geographical origin, host species, and year. To identify the most significant factor, we used multidimensional scaling (MDS). The results showed clear clustering by geographical origin, with three distinct clusters formed by accessions from the Netherlands and other neighboring European countries, including Belgium (Figure 10A). When the MDS scatter plot was color-coded by host (Figure 10B), most accessions were derived from *Solanum* (tomato, shown in blue), with fewer from *Capsicum* sp. (pepper). No distinct clustering by host was observed, likely due to the dominance of tomato isolates, suggesting that host species is not a major driver of diversity. In contrast, when color-coded by collection year (Figure 10C), accessions from the same year tend to cluster together, supporting the hypothesis that the ToBRFV is not evolving rapidly within the same geographical region.

## 4. Discussion

Published analyses of *tobamovirus* isolates from different geographical regions and individual species suggest that these viruses exhibit limited genetic diversity across their genomes [22,49]. In contrast with this observation, the emergence of new strains and even new species [24,50] shows that tobamoviruses are actively mutating and evolving. The most recent species in the genus is *T. fructirugosum,* which affects global tomato production. A notable feature of *T. fructirugosum* is that it broke genetic resistance based on the *Tm-2^2^* gene, which had been effective for 60 years against tobamoviruses, including *T. tomatotessellati* and *T. tabaci* [51,52]. The discrepancy between low genetic diversity in some *Tobamovirus* species and the emergence of new strains and species highlights the need to better understand genomic variation and its relationship to *Tobamovirus* evolution.

In RNA viruses, low genetic diversity correlates with genetic stability [42]. In *T. fructirugosum*, similar resistance-breaking mutations occurred independently twice in two different strains (Figure 8) [47,48]. In a genome that is 6400 nt long, the random probability of two single-nucleotide mutations occurring at the position is 2.4 × 10^−8^. This indicates that the genetic stability of tobamoviruses (Figure 4) is not the result of the absence of mutations (Figure 8A). Instead, it may indicate strong purifying selection (Figure 5D), low positive selection (Figure 5E) and the evolutionary pressure to preserve essential protein functions. Despite these restrictions, as in the case of ToBRFV, in tobamoviruses, RNA recombination followed by single-nucleotide and single-amino-acid substitutions can give rise to strains or species [4,53].

After measuring nucleotide variation for the entire genome and for all members of the genus, our results showed that tobamoviruses exhibit low genetic diversity and are under strong negative selection (Figure 5). On average, tobamoviruses harbor nucleotide diversity in 11% of the nucleotide positions on the genome. This is 4.8 times lower than that observed for *Potyvirus sacchari* (Figure 3), 2.3 times lower than that observed for poleroviruses [28], 3.3 times lower than that observed for orthotospoviruses [33], and 2.6 times lower than that observed for betacoronaviruses [54]. *Tobamovirus* variation was approximately the same as that observed in *M. zeae* (Figure 3), a virus considered genetically stable [42]. In contrast to what was observed in potyviruses [31], orthotospoviruses [33], poleroviruses [28], and betacoronaviruses [54], tobamoviruses do not harbor hypervariable areas (Figure 7 and Figure 8).

Tobamoviruses have been evolving alongside their eudicotyledonous hosts since their establishment in the asterid, rosid, and caryophyllid lineages approximately 112.9 million years ago [55]. Currently, tobamoviruses infect hosts across eight botanical families (Figure 2), and our phylogenetic analyses showed that tobamoviruses cluster according to the botanical family of their host. Fifteen virus species that infect plants in the family *Solanaceae* form a clade, while viruses infecting plants in the family *Cucurbitacea* form a separate clade (Figure 2). This pattern of host-specific clustering supports the hypothesis of long-term co-evolution, adaptation, and specificity of viruses in general, and tobamoviruses in particular, to their host plants [24].

The low genomic variation observed in tobamoviruses in this study (Figure 3 and Figure 4) contrasts with previous findings that reported a rapid evolutionary rate in this genus [24]. In that previous study, Bayesian coalescent methods were used to analyze nucleotide substitutions, and the results showed that the substitution rate is similar to that observed in animal and plant RNA viruses, and the authors concluded that *T. tomatotessellati* exhibits rapid evolutionary rates [24]. Our findings are consistent with high mutation rate under strong purifying selection (Figure 5D), low positive selection (Figure 5E) and the evolutionary pressure to preserve essential protein functions [56].

To further explore mutation distribution, we analyzed the five most variable tobamoviruses. Across the genome, nucleotide substitutions accumulated at relatively similar rates across open reading frames (ORFs) (Figure 5A). The most variable species, *T. kyuri*, exhibited a region within the 183 kDa replicase ORF where mutations accumulated at levels exceeding the genome-wide average (Figure 6). Similarly, *T. plantagonis* showed a distinct mutation accumulation region in the 183 kDa replicase ORF (Figure 7). In contrast, the other three viral species displayed overall low variation with no specific regions of concentrated mutations.

For the five most variable viruses and *T. fructirugosum*, ORFs are predominantly under negative selection, as the number of negatively selected sites normalized to ORF length exceeds that of positively selected sites (Figure 5). Additionally, the randomly expected number of sites under negative selection is significantly higher than that observed, further supporting the low genomic variation rate in tobamoviruses. Fewer sites under purifying selection than expected suggest relaxed functional constraint or tolerance of variation in this region [37]. This is consistent with previous analyses of *T. fructirugosum* [57,58], and can be explained by the model that strains harboring deleterious mutations are unlikely to be fixed in the population [59] and are thus not present in the sequence dataset.

Despite low genetic variation, there is evidence of adaptive evolution in *T. mititessellati*, *T. tabaci*, *T. capsica,* and *T. youcai* [22,56]. For instance, a single-nucleotide change in the overlapping of MP and CP open reading frames resulted in altered symptoms in *T. youcai* [60]. Furthermore, the low genomic variation and negative selection pressure observed in tobamoviruses do not preclude their ability to adapt and overcome host resistance. In tomato, resistance against tobamoviruses is conferred by *Tm*-1, *Tm*-2, and *Tm*-2^2^ [23]. However, the first documented case of resistance breaking occurred in 2014 in Israel, where *T. fructirugosum* (ToBRFV) successfully infected tomato and pepper [3], and infected tomato in the greenhouse during the spring of 2015 in Jordan [4]. A notable example of resistance breakdown is strain ToBRFV-Tom2M-Jo-MZ2438228.1, where resistance was overcome due to two amino acid substitutions in the movement protein at amino acids 22 (Phe replaced by Tyr) and 82 (Asn replaced by Lys) [47]. These two amino acid changes resulted from single-nucleotide substitutions [57,58] at positions 4975 and 5156, respectively (T replaced by A in both cases) [47]. More recently, on a second strain (ToBRFV_G78_RB-OR760199), a single amino acid substitution (Asn82Lys), resulting from a single-nucleotide substitution at position 5156 (T is replaced by G) in the movement protein, also broke resistance [48]. This site maps to an area with a small variation peak that is not maintained in other relative species (Figure 8). These observations support the hypothesis that tobamoviruses can mutate and evolve even with limited variation in nucleotides and highlight the need to better understand interactions between plant and viral proteins [61].

The emergence of *T. fructirugosum* is linked to a recombination event between *T. tomatotessellati* and *T. tabaci* [4,53]. After the recombination event, one or two mutations in the movement protein were identified as responsible for overcoming *Tm*-2^2^ resistance [51,52,62]. Since RNA recombination requires co-infection of the same plant, and even the same cell [59,63], it is unlikely that recombination between *T. tomatotessellati* and *T. tabaci* occurred in commercial tomato. It is most likely that the recombination event occurred in an alternate host, such as wild tomato, breeding lines, or other species that serve as a host for both *T. tomatotessellati* and *T. tabaci.* In this scenario, following the recombination event, the commercial tomato imposed selection pressure, allowing only the strain capable of breaking resistance to infect, replicate, and spread. The high success rate of *T. fructirugosum* is not due to its higher nucleotide diversity (Figure 4) or its ability to adapt to different hosts. Instead, it could be explained by the uniform genetics of commercial tomato cultivars, which, for the last sixty years, were bred to harbor *Tm*2^2^-mediated resistance against tobamoviruses [3,4]. In this model, breeding for resistance to *T. fructirugosum* is likely to resemble *Tm*2^2^-mediated resistance to tobamoviruses: initially effective against circulating strains but with the potential for resistance-breaking strains to arise through recombination, mutation, and selection in alternative hosts.

To better understand the factors influencing genomic diversity in *T. fructirugosum*, we generated a phylogenetic tree relating geographical distribution, host plant, and date of collection to the diversity observed in this species (Figure 9). Our phylogenetic analysis revealed that *T. fructirugosum* isolates cluster into four major groups that could correlate with the host, country of origin, or year of collection. Accessions from alternative hosts, such as *Capsicum* spp., clustered separately, indicating the emergence of variation associated with host adaptation. Multidimensional scaling analysis illustrated the formation of three distinct clusters, primarily explained by geographical origin and including accessions from the Netherlands and Belgium. While geographical separation played a significant role in defining viral clusters, the host and year of collection had a minor impact (Figure 10). Clusters of isolates from the same collection year imply that *T. fructirugosum* does not undergo rapid mutation within localized outbreaks. This is consistent with previous reports indicating that *T. fructirugosum* exhibits a relatively low evolutionary rate compared to other plant RNA viruses [21,57].

The results described here show that low genetic variation, strong negative selection, and the absence of hypervariable areas are general features of the genus *Tobamovirus*. The emergence of new strains and species, such as *T. fructirugosum*, implicates alternative hosts as a source of genetic variation that is later selected and influenced by geographical distribution more than host specificity. Accordingly, these results highlight the importance of minimizing *tobamovirus* spread by monitoring global seed trade.

## Figures and Tables

**Table 1:** *Tobamovirus* species downloaded from NCBI and the number of accessions used in this analysis.

Species Name	Common Name	ICTV Abbreviation	No. of Accessions ^1^	Reference Genome ^2^	Length (nt)	95% Length	Accessions (≥95%) ^3^
*T. maculacapsici*	Bell pepper mottle virus	BPMV	5	NC 009642.1	6375	6056	4
*T. brugmansiae*	Brugmansia mild mottle virus	BrMMV	2	NC 010944.1	6381	6062	2
*T. cacti*	Cactus mild mottle virus	CMMoV	5	NC 011803.1	6449	6127	3
NA ^4^	Cactus tobamovirus 1		2	MW938767.1	6458	6135	2
NA ^4^	Cactus tobamovirus 2		2	MW938766.1	6368	6050	2
NA ^4^	Chili pepper mild mottle virus		14	MN164455.1	6383	6064	2
*T. clitoriae*	Clitoria yellow mottle virus	CliYMV	2	NC 016519.1	6514	6188	2
*T. maculafructi*	Cucumber fruit mottle mosaic virus	CFMMV	19	NC 002633.1	6562	6234	4
*T. viridimaculae*	Cucumber green mottle mosaic virus	CGMMV	484	NC 001801.1	6424	6103	185
*T. cucumeris*	Cucumber mottle virus	CMoV	2	NC 008614.1	6485	6161	2
*T. frangipani*	Frangipani mosaic virus	FrMV	9	NC 014546.1	6643	6311	4
*T. fortpiercense*	Hibiscus latent Fort Pierce virus	HLFPV	26	NC 025381.1	6431	6109	10
*T. singaporense*	Hibiscus latent Singapore virus	HLSV	9	NC 008310.2	6485	6161	6
NA ^4^	Hoya chlorotic spot virus		2	NC 034509.1	6386	6067	2
NA ^4^	Hoya necrotic spot virus		4	LC807720.1	6425	6104	3
*T. kyuri*	Kyuri green mottle mosaic virus	KGMMV	51	NC 003610.1	6514	6188	4
*T. maracujae*	Maracuja mosaic virus	MarMV	2	NC 008716.1	6794	6454	2
*T. obudae*	Obuda pepper virus	ObPV	4	NC 003852.1	6507	6182	4
*T. odontoglossi*	Odontoglossum ringspot virus	ORSV	241	NC 001728.1	6618	6287	13
NA ^4^	Opuntia virus 2		19	NC 040685.2	6453	6130	5
*T. paprikae*	Paprika mild mottle virus	PaMMV	19	NC 004106.1	6524	6198	8
*T. passiflorae*	Passion fruit mosaic virus	PFMV	2	NC 015552.1	6791	6451	2
*T. capsici*	Pepper mild mottle virus	PMMoV	662	NC 003630.1	6357	6039	77
NA ^4^	Piper chlorosis virus		3	ON924221.1	6237	5925	2
*T. plumeriae*	Plumeria mosaic virus	PluMV	4	NC 026816.1	6688	6354	4
*T. muricaudae*	Rattail cactus necrosis-associated virus	RCNaV	22	NC 016442.1	6506	6181	5
*T. rehmanniae*	Rehmannia mosaic virus	RheMV	75	NC 009041.1	6395	6075	8
*T. plantagonis*	Ribgrass mosaic virus	RMV	41	NC 002792.2	6311	5995	11
*T. streptocarpi*	Streptocarpus flower break virus	SFBV	6	NC 008365.1	6279	5965	4
*T. mititessellati*	Tobacco mild green mosaic virus	TMGMV	398	NC 001556.1	6355	6037	36
*T. tabaci*	Tobacco mosaic virus	TMV	653	NC 001367.1	6395	6075	94
*T. fructirugosum*	Tomato brown rugose fruit virus	TBRFV	451	NC 028478.1	6393	6073	232
*T. tomatotessellati*	Tomato mosaic virus	ToMV	372	NC 002692.1	6383	6064	86
*T. maculatessellati*	Tomato mottle mosaic virus	ToMMV	62	NC 022230.1	6398	6078	26
*T. tropici*	Tropical soda apple mosaic virus	TSAMV	13	NC 030229.1	6350	6033	11
*T. rapae*	Turnip vein clearing virus	TVCV	29	NC 001873.1	6311	5995	10
*T. wasabi*	Wasabi mottle virus	WMoV	6	NC 003355.1	6298	5983	6
NA ^4^	Watermelon green mottle mosaic virus	WGMMV	6	MH837097.1	6482	6158	6
*T. anthocercis*	Yellow tailflower mild mottle virus	YTMMV	97	NC 022801.1	6379	6060	4
*T. youcai*	Youcai mosaic virus	YoMV	129	NC 004422.1	6303	5988	30
*T. cucurbitae*	Zucchini green mottle mosaic virus	ZGMMV	10	NC 003878.1	6513	6187	5

^1^ All available nucleotide accessions in NCBI for each viral species. ^2^ Accession number used as a reference for each species. ^3^ Number of accessions with at least 95% the length of the reference genome. ^4^ Indicates virus name in NCBI that is not listed in ICTV.

## Data Availability

The in-house Python 3.12.5, Bash 5.2, and R scripts used in this analysis are available upon request.

## Supplementary Materials

The following supporting information can be downloaded at https://www.mdpi.com/article/10.3390/v17091284/s1: Table S1: NCBI accessions of each *Tobamovirus* species used in this study. Table S2: tomato brown rugose fruit virus (ToBRFV) NCBI accessions used in this study.
